# Glances at the Hospitals

**Published:** 1902-07-26

**Authors:** 


### GLANCES AT THE HOSPITALS.

### ROYAL INFIRMARY, DUNDEE.

THE ordinary income for the year was £9,955, and the ordinary expenditure was £10,223. Structural alterations and improvements cost £1,139, so that at the end of the year there was an adverse balance of £1,406. At the beginning of the year there were in residence 215 patients; 3,041 were admitted during the year, making a total of 3,256. Of these 2,580 were discharged cured or relieved, 208 not relieved, 244 died, and 224 remained in the infirmary. In addition to these there were 10 maternity cases, and 10,014 out-patients. The average daily number resident was 208; the highest number at any one time was 244, and the lowest was 170. The average of each patient's stay in the hospital was 23 days. The death rate was 7·49 per cent., but if cases dying within 48 hours of admission be excluded, it would be only 5·8 per cent. We are pleased to note that one of the resident medical officers is a lady. The medical and surgical statistical tables show the sex of the patients, the numbers treated for the various diseases, the numbers cured, relieved, or died, the number of days in the hospital, and there is another column for special remarks on the cases. There were 72 cases of acute bronchitis treated, with one death, and the average number of days in the hospital was 15½. Of lobar pneumonia there were 71 cases, with 31 deaths, the average duration of treatment in the recovered cases was 25½ days. The operations table shows that a large number of operations were performed during the year, but oddly enough the results are not stated. The anæsthetic table shows that chloroform was administered 1,170 times, ether 13 times, a mixture of ether and chloroform 4 times, and the A.C.E. mixture once.

### YORK COUNTY HOSPITAL.

The receipts were, including legacies, £6,567, and the expenditure was £6,739. The figures show that the ordinary income was £1,391 below the ordinary expenditure. The year began with an adverse balance of £2,453, and it closed with an adverse balance of £2,624. The expenditure was £645 higher than it was in the previous year, but this was chiefly owing to the increased number of patients, the daily average having gone up from 107 to 117, and in addition to this the out-patients numbered 9,924, which was fully 2,000 more than in the previous year. To meet the serious deficiency in the income it was decided to hold a house-to-house canvass in the city and neighbourhood. On referring to the statement of income we found that the workmen's and miscellaneous collections amounted only to £411. We believe that this might be greatly increased. The total number of in-patients was 1.217. Of these 950 were discharged cured or relieved, 25 not relieved, 117 died, and 125 remained under treatment. The average stay in the hospital was 36 days. This is a long time, and undoubtedly some economy would result if it were reduced to 24 days. The death-rate was 9·6 per cent., but 32 died within 48 hours of admission, and 2 were brought in dead. The tables give the number of cases treated and the numbers dying of the various diseases. Of 35 cases of pneumonia, 6 died; of 59 cases of enteric fever, 13 died; of 11 operations for strangulated hernia all were successful, and the same result followed in 10 operations for the radical cure of hernia. The anæsthetic table shows that chloroform was the agent in 135 instances, ether in 209, A.C.E. mixture in 145, nitrous oxide in 113, and A.C.E. mixture and ether in 45.

### SOUTHAMPTON HOSPITAL.

The total ordinary income was £5,015, and the total ordinary expenditure was £6,740, showing an excess of expenditure over ordinary income of £1,725, but the total income from all sources was £6,662, and the total expenditure £7,588. The committee point out that a debt of £8,000 remains on the building fund, and that until this is paid off the income will be still further crippled as interest must be paid on the £8,000. It is not without reason, therefore, that the committee plead for new and larger subscriptions. The workmen's donations and subscriptions have increased somewhat, but are still only £162, a sum far below what we should expect to find in Southampton and neighbourhood. At the beginning of the year the hospital contained 66 patients, 1,158 were admitted, 23 readmitted, making a total of 1,247 under treatment. Of these 784 were discharged cured, 187 relieved, 52 for various reasons, 103 died, and 98 remained in the hospital. The average daily number resident was 81, the average stay in the hospital was 23 days, the cost of each bed occupied was £72, the cost per patient was £4 14s., and the cost of each patient per day was 3s. 11d. The total number of out-patients was 4,707. The medical and surgical tables are so meagre that no useful lesson can be got from them, but the table of operations gives results. We note that ovariotomy was performed twice, both cases recovering; the operation for the radical cure of hernia, 24 times, recovery following in all cases. There were 41 amputations, death following in three, the fatal cases being thigh, hip-joint, and shoulder joint. The total number of operations was 769. There is no anæsthetic table.

### ROYAL HOSPITAL, BATH.

At the beginning of the year 1900 there was an adverse balance of £1,324 due to the bank, and by the end of the year this had increased to £3,559. From the Governors' report it would seem that for many years the income has fallen short of the expenditure, but that legacies have been used to tide over these financial difficulties instead of being invested as a source of permanent income. As this permanent income is only £662 a year, and as £6,500 a year is required to meet the working expenses, it is clear that the former has been sacrificed to the latter. The Governors add that things have now come to a crisis, and indeed it is evident that either the expenses must be cut down or the annual subscriptions increased. On the 1st of January the hospital contained 81 patients, and 1,303 were admitted during the year, making a total of 1,384. Of these, 1,070 were discharged recovered or relieved; 64 not relieved, and 87 died; leaving 82 in residence at the end of the year. The out-patients numbered 8,608. The medical and surgical statistics are very clearly arranged, and they convey sufficient information for all purposes of comparison with other institutions. There were 14 cases of enteric fever, of whom 11 recovered, 1 died, and 2 were under treatment on the date of the report. There were 25 cases of pneumonia with 5 deaths. It is curious that the operation table gives no results. The operation for the radical cure of hernia was performed 18 times; trephining the skull 3 times; ovariotomy 3 times. It would have been interesting to know the results in these and in many other operations. There is an anæsthetic table which tells us that gas was administered 300 times; gas and ether 228 times; chloroform 101 times; chloroform and A. C. E. mixture 101 times, and ether 27 times.

### EDINBURGH ROYAL INFIRMARY.

At the beginning of the year this hospital contained 655 patients, and 8,914 were admitted during the year making a total of 9,569 patients during the year. The average daily number resident was 701. The average periods of residence were:—in the medical wards, 28 days; in the surgical wards, 25 days, and in the Lock wards 21 days. The percentage of deaths was 8; but if cases dying within 48 hours of admission be omitted, the percentage falls to 5·7. The ordinary expenditure per bed occupied was £68 5s., but this includes the cost of the Out-patients' Department. The Nurses' Training School of this Infirmary is famous throughout Scotland, and the matrons of 39 Scotch institutions were trained here; as were also the matron of the Stanley Hospital, Liverpool, and of the Nursing Home, Swansea. The staff numbered 195, and during the year there were 670 applications for admission as against 578 in the previous year. The report of the Medical Registrar shows that of the 4,625 cases treated during the year, 1,421 recovered; 1,891 were relieved; 412 not relieved; discharged for other reasons, 155; died, 481. The tables have been prepared with the greatest care. To the surgical wards, 4,789 cases were admitted, and the death-rate was 5·7 per cent. The total number of operations performed was 2,862, and the average mortality was 5·2 per cent. The operation table, like the others, gives every information required.

### CUMBERLAND INFIRMARY.

The receipts amounted to £5,433 and the expenditure to £4,931. The total indebtedness to the bank and other creditors was £1,476, being nearly £100 less than it was for the previous year. The average annual cost of maintenance per patient was £19 14s., and the cost per bed occupied was £56 12s. There were 70 patients in residence at the beginning of the year, and 818 were admitted during it. Of these 718 were discharged recovered or relieved, 40 were incurable, 49 died, and 81 remained in the hospital. The average stay in the hospital was 33 days. The medical and surgical tables have separate columns showing the numbers suffering from the various diseases, and the numbers cured, relieved, or died. There is also a table showing the operations and the results; but no anæsthetic table is given.

